# AngioJet Rheolytic Thrombectomy to Treat Inferior Vena Cava Filter-Related Thrombosis: Efficacy and Safety Compared With Large-Lumen Catheter Suction

**DOI:** 10.3389/fcvm.2022.837455

**Published:** 2022-03-21

**Authors:** Zhengli Liu, Guanqi Fu, Maofeng Gong, Boxiang Zhao, Jianping Gu, Tao Wang, Yangyi Zhou, Xu He, Jie Kong

**Affiliations:** Department of Interventional Radiology, Nanjing First Hospital, Nanjing Medical University, Nanjing, China

**Keywords:** thrombosis, inferior vena cava filter, interventional therapy, percutaneous large-lumen catheter suction, Angiojet rheolytic thrombectomy

## Abstract

**Objective:**

To compare the efficacy and safety of AngioJet rheolytic thrombectomy (ART) and large-lumen catheter suction (LCS) in the treatment of inferior vena cava (IVC) filter related IVC-iliac vein thrombosis.

**Methods:**

The clinical data and medical imaging materials of 65 cases were collected, which suffered acute inferior vena cava filter related IVC-iliac vein thrombosis and received percutaneous mechanic thrombectomy (PMT) from June 2016 to June 2020 in our center, including 32 cases of LCS group and 33 cases of ART group. The final thrombolysis rate, the incidence of complications, and the follow-up are evaluated.

**Results:**

The limb swelling was significantly relieved in patients with PMT after treatment. The peri-diameter difference of the limb in the LCS group before and after treatment was [(5.20 ± 2.03) vs. (2.17 ± 1.29) cm, *P* < 0.05], and that in the ART group before and after treatment was [(4.79 ± 2.23) vs. (1.74 ± 0.94) cm, *P* < 0.05]. The amount of postoperative recombinant tissue-type plasminogen activator (rt-PA) is reduced in ART group [(57.97 ± 21.25) in LCS group vs. (40.45 ± 20.89) mg in ART group, *P* < 0.05], and the thrombolysis rate was higher than that of the LCS group [(74.13 ± 19.74% in LCS group) vs. (84.58 ± 11.90% in ART group %), *P* < 0.05]. No serious complications occurred during the treatment.

**Conclusion:**

Both LCS group and ART group have good thrombosis clearance effects on the inferior vena cava filter related IVC-iliac vein thrombosis. ART can reduce the rt-PA dose, increase the thrombolysis rate and reduce the risk of bleeding during thrombolysis.

## Introduction

Deep vein thrombosis (DVT) is a type of disease caused by abnormal agglutination of blood in the deep veins of the lower extremities. It often manifests as swelling, pain, and dysfunction of the affected limb. If DVT of the lower extremity is not treated in time, 50–60% of the patients can merge pulmonary embolism (PE), and severe cases can cause death. PE and DVT are collectively referred to as venous thromboembolism (VTE). The average annual incidence rate of VTE in the United States is 548 thousand. The incidence of VTE in the European population is estimated at 104 ~ 183/10 million (1). The inferior vena cava filter (IVCF) can effectively intercept thrombus, thereby reducing the occurrence of fatal PE (2). However, in recent years, with the widespread use of IVCF, the incidence of inferior vena cava filter-related thrombosis has increased. The main causes include secondary thrombosis such as the inferior vena cava filter to capture and fall off the thrombus, non-standard anticoagulation treatment, filter-related intimal injury, and another secondary thrombosis. Catheter-directed thrombolysis (CDT) is an effective method for filter-related inferior vena cava-iliac vein thrombosis, which can reduce the incidence of posttrombosis syndrome (PTS) (3). However, prolonged use of thrombolytic therapy will increase the risk of bleeding. In contrast, percutaneous mechanic thrombectomy (PMT) can quickly and directly reduce the volume of thrombus, and it can achieve better results for filter-related inferior vena cava-iliac vein thrombosis, which has poor efficacy in CDT (4). PMT includes the use of thrombus removal devices to remove thrombus and the use of large-lumen catheters to aspirate thrombus, but there is still a lack of comparative studies on which method is better. Our center researched and compared the clinical and imaging data of 65 patients with acute inferior vena cava-iliac filter-derived thrombosis undergoing PMT.

## Materials and Methods

### Basic Information

The study was approved by the ethics committee of our hospital (KY20210609-15) and by the written consent of patients. A retrospective analysis contained patients with acute inferior vena cava-iliac filter-derived thrombosis admitted to our hospital from June 2016 to June 2020, including 30 males and 35 females; aged 17–82 years old (57.82 ± 19.17 years). Among the 65 patients, 18 had a history of injuries and fractures, seven had a history of pregnancy and postpartum, nine had a history of venous thromboembolism, seven cases had a history of stroke or paralysis, five had a history of malignancy of various organs of the whole body, and 19 had no obvious cause. All patients underwent color Doppler ultrasound, venography or CTV to confirm iliac vein thrombosis of the lower extremities (central or mixed), and the inferior vena cava filter was placed to prevent fatal pulmonary embolism. No inferior vena cava thrombosis was found on inferior vena cava angiography when the filter was placed. All patients had received CDT treatment for more than 3 days. After the treatment, there were still a large amount of thrombus remaining in the inferior vena cava-iliac vein, followed by percutaneous mechanic thrombectomy treatment in our center, including 32 cases in the LCS group and 33 cases in the ART group. All the patients in this study were given informed consent to the diagnosis and treatment process. The baseline data of the patients were listed in Table 1.

**Table 1:** Basic information of the two groups of patients.

**Patient info**	**LCS group**	**ART group**
*n*	32	33
Gender (M/F)	16/16	14/19
Age	59.34 ± 17.38	56.70 ± 19.28
Onset time (d)	6.19 ± 3.07	6.18 ± 6.49
Whether with PE (Yes/No)	14/18	12/21
Preoperative diameter difference	5.20 ± 2.03	4.79 ± 2.23
**Thrombosis inducement (** ***n*** **)**		
Injury and fracture	11	7
Pregnancy and postpartum	2	5
History of venous Thromboembolism	4	5
Malignancy	3	2
VNo obvious inducement	9	10

### Surgical Methods

For patients with filter-related inferior vena cava-iliac vein thrombosis, after 3 days of CDT treatment, the thrombus was not significantly improved, and PMT was used to treat residual thrombosis. The contralateral femoral vein was the preferred puncture route. When the occluded segment of thrombus was difficult to open, a trans-jugular route or popliteal vein was used. We used a common guide wire (Terumo, Japan) with a 4F single-curved catheter to pass through the thrombosis occluded segment. If it was difficult to pass, we used a Cobra catheter (Cordis, United States) with an exchange guide wire to try to pass the thrombo-occluded segment. A thrombolytic catheter (Merit, USA) was used to determine the position and length of the thrombotic occlusion segment.

**Figure 1:**
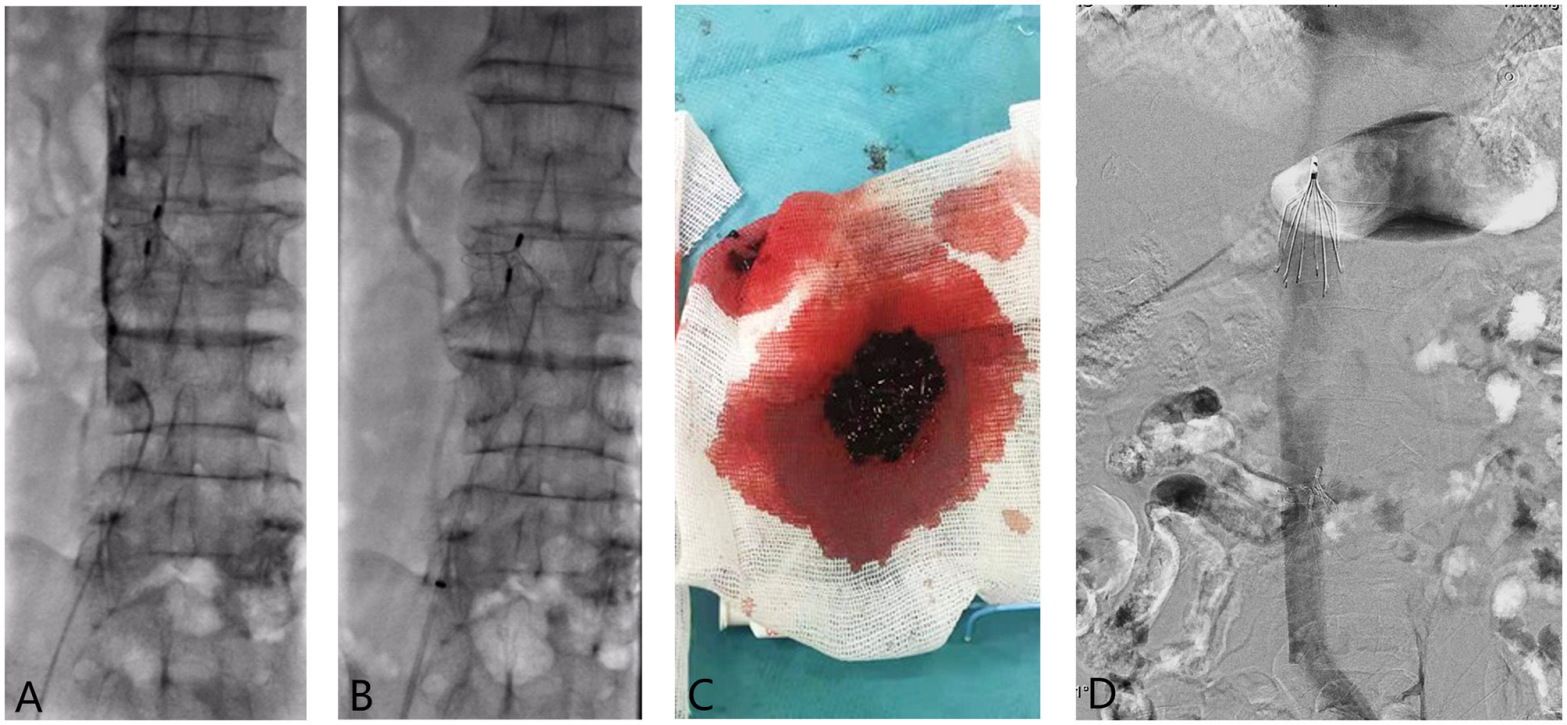
**(A–D)** A 80-year-old patient was placed a filter (Simon) due to deep venous thrombosis of the right lower limb in 2005. After the filter was placed, the patient stopped taking warfarin orally for 2 years. The patient had swelling of both lower limbs 5 days ago, and a Denali filter was implanted in the other hospital. After the operation, he was treated with 250,000 IU/qd urokinase for thrombolysis via the dorsal vein of the foot, while the thrombus treatment effect of the patient was not satisfactory. So he was admitted to our department for further treatment. **(A)** Preoperative angiography showed massive thrombosis blow the IVCF, and hand-push angiography showed obvious “dual-track sign” of thrombosis; **(B)** The 8F Guiding catheter was placed under negative pressure while withdrawing the catheter and sucking the thrombus until exiting the body. We repeatedly sucked the thrombus a total of about three times; **(C)** A large number of thrombus withdrawn from the large lumen catheter during the operation; **(D)** After CDT, the angiography showed that the inferior vena cava was unobstructed; and the IVCF was taken out.

Patients in the LCS group were introduced to the inferior vena cava filter through a 10F vascular sheath (Cordis, United States) and a 6F/8F large-lumen catheter (Boston Scientific, United States) guided by an exchange guidewire (Terumo, Japan). At the lower position of thrombus, we withdrew the exchange guide wire, connected a 50 ml injector to the end of the large lumen catheter, and kept the negative pressure of the syringe constant for thrombus suction. Then we withdrew the large-lumen catheter while aspirating, and rotated the tip of the large-lumen catheter at the same time, so as to withdraw while rotating. If the angiography showed that the thrombus still remained, we repeated the LCS 2–3 times. Then the large lumen catheter was withdrawn, and the 4F pigtail catheter (Cordis, USA) was replaced with the inferior vena cava angiography to evaluate the patency of the inferior vena cava-iliac vein. The treatment process of typical LCS method is shown in Figures 1A–D.

**Figure 2:**
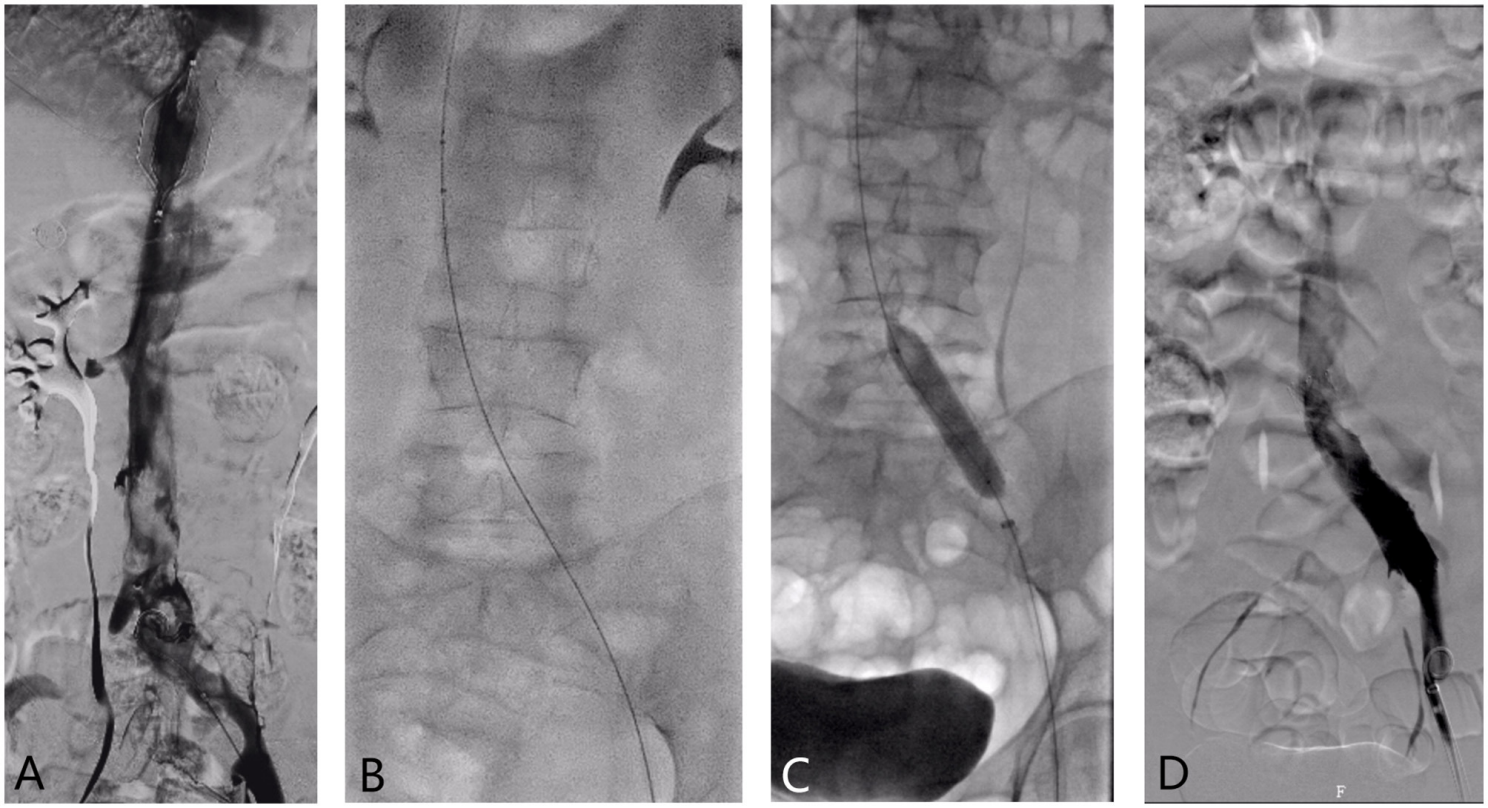
**(A–D)** The 59-year-old female patient was admitted to the hospital with “left lower limb swelling for more than 6 days after meniscus injury”. An IVCF was placed in the other hospital. **(A)** Inferior vena cava angiography showed a large amount of thrombosis below the filter and left iliac vein compression; **(B)** The AngioJet rheolytic thrombus removal device was used for iliac vein-inferior vena cava thrombosis suction; **(C)** Balloon dilatation was performed on the narrow left iliac vein. **(D)** Angiographic reexamination was performed after thrombus was removed by ART, suggesting that the iliac vein-inferior vena cava thrombosis was significantly improved than before.

After exchanging guidewires to reach the position of the inferior vena cava thrombus in the ART group, we first used the inject mode of the AngioJet rheolytic thrombus removal device (Medrad, USA) to inject 250,000 units of urokinase (dwassolved in 250 ml of normal saline). After waiting for 15 min, we changed the injection mode to the thrombus suction mode, and moved the thrombus suction catheter (Boston Scientific, USA) along the exchange guide wire at a speed of 1 mm/s to aspirate the thrombus. If the angiography showed that the thrombus still remains, we repeated the suction 2–3 times. After the suction was completed, the thrombus suction catheter was withdrawn, and the 4F pigtail catheter was replaced to perform the inferior vena cava angiography to assess the patency of the inferior vena cava-iliac vein. The treatment process of typical ART method is shown in Figures 2A–D. The patient's treatment status was listed in Table 2.

**Table 2:** Treatment of patients.

**Group**	**Induced filter-type (** ***n*** **)**					**Thrombolytic time (d)**	**Dose of rt-PA (mg)**	**Length of stay (d)**
	**Aegisy**	**Optease**	**Denali**	**LP**	**Simon**			
LCS	12	9	8	2	1	4.25 ± 1.59	57.97 ± 21.25	10.66 ± 3.69
ART	11	11	6	3	2	4.06 ± 1.71	40.45 ± 20.89	9.61 ± 3.04
*t* or χ^2^	-	-	-	-	-	0.462	3.351	1.253
*P*-value	-	-	-	-	-	0.646	0.001	0.215

### Postoperative Treatment and Follow-Up Methods

During hospitalization, the patient received anticoagulant therapy with low molecular weight heparin sodium (Italy, ALFA WASSERMANN Inc). The dosage was 4250IU, subcutaneously injected twice a day. All patients received transcatheter recombinant tissue-type plasminogen activator (rt-PA) thrombolysis after operation, and the postoperative rt-PA dose was 10–20 mg/d. 3 days after the operation, the venography was rechecked to observe the recovery of the thrombus. If the thrombus was improved, the thrombolytic catheter was removed, and the standard anticoagulation was continued for 2–3 days before being discharged from the hospital. If the thrombus still remained, we continued to use rt-PA, but the total thrombolysis time shall not exceed 1 week. After discharge, the patients were treated with oral rivaroxaban 20 mg/QD. Patients were followed up at 1, 3, 6, 12 months after surgery and once a year after that. Evaluation methods included six routine blood coagulation, intravenous ultrasound and contrast examinations, and assessment of venous thrombosis and valve function. Anticoagulant was taken orally for at least 3 months, and the dosage and time of anticoagulant should be adjusted according to the recovery of thrombus.

### Efficacy Evaluation

The evaluation of curative effect in this study was mainly divided into the following aspects (5): (1) Calculated the circumference difference to evaluate the swelling rate of the limbs, and the degree of swelling relief of the limbs. The peripheral diameter difference between the healthy thigh and the affected thigh at 15 cm above the patella was measured before and after operation. (2) Calculated the thrombus clearance rate according to the results of the angiographic examination, according to Porter et al. The thrombus clearance score proposed by humans divided the deep veins of the lower extremities into seven segments. Each segment of completely patency vein was scored as 0 points, partial patency scored as one point, and blocked as two points, and then calculated venous patency rate = (pre-thrombolysis score-dissolution score after thrombolysis)/Score before thrombolysis ×100%. Thrombus clearance rate >90% was defined as grade III, 50–90% as grade II, and <50% as grade I (6). (3) The patients would be followed up at 1, 3, 6, and 12 months after the operation. Subjective symptoms included pain, muscle cramps, heaviness, paresthesia, and itching. Objective signs included pretibial edema, pigmentation, varicose veins, and skin tone. Six cases suffered redness, skin hardening, and calf squeezing pain. Color Doppler ultrasound and angiography of the deep veins of the lower extremities were performed to assess the general condition of the deep veins of the lower extremities and valve function.

### Safety Evaluation

By comparing the occurrence of severe complications during hospitalization, the occurrence of new symptomatic pulmonary embolism, the presence and the location of hemorrhage and the occurrence of hemoglobin decline, the safety evaluation of the two methods of thrombus removal was carried out. Among them, serious complications were defined as hemoglobin decrease >20 g/L and hemorrhage that requires infusion of ≥0.4 L suspended red blood cells, intracranial hemorrhage or other important organ hemorrhage, and death.

### Statistical Methods

SPSS 22.0 software was used to evaluate the clinical efficacy of the two mechanical thrombus removal methods. We used the Wilcoxon-rank test of paired samples, the Fisher chi-square test and independent sample *t*-test to analyze the differences between the LCS group and the ART group. *P* <0.05 was considered of statistically significance.

## Results

### Evaluation of Efficacy

The LCS group and the ART group were evaluated for the degree of thrombus clearance immediately after the thrombus was cleared. Of the 32 patients in the LCS group, eight patients reach the thrombus clearance of grade III, 19 patients of grade II, and five patients of grade I. The thrombus clearance of 33 patients in the ART group all reached grade II/ III, including 13 cases of grade III and 20 cases of grade II. The swelling of all patients after PMT treatment was relieved compared with that before operation. The diameter difference of 15 cm above the suprapatella was recorded before and after the operation. The diameter difference of the bilateral suprapatella was recorded as well. The LCS group was (5.20 ± 2.03) cm and (2.17 ± 1.29) cm, the ART group was (4.79 ± 2.23) cm and (1.74 ± 0.94) cm, the differences before and after treatment were statistically significant (*P* < 0.05). There was no statistical significance in terms of thrombolysis time, difference of 15 cm above patella before and after treatment of the two groups. However, the postoperative rt-PA dose required for the ART group was significantly reduced in the larger lumen catheter suction group (*P* < 0.05), the thrombus clearance rate of the ART group was better than that of the large lumen catheter suction group (*P* < 0.05). The comparison of curative effect of two groups of treatment methods is shown in Table 3.

**Table 3:** Comparison of curative effect of two groups of treatment methods.

**Group**	**Thrombus clearance grade (** ***n*** **/%)**			**Thrombus clearance rate (%)**	**Preoperative diameter difference (cm)**	**Postoperative diameter difference (cm)**
	**I**	**II**	**III**			
LCS	5 (15.63)	19 (57.58)	8 (25.00)	74.13 ± 19.74	5.20 ± 2.03	2.17 ± 1.29
ART	0	20 (60.61)	13 (39.39)	84.58 ± 11.90	4.79 ± 2.23	1.74 ± 0.94
*t* or χ^2^	6.202			−2.576	0.784	1.533
*P*-value	0.045			0.013	0.436	0.130

### Safety Evaluation

There were no serious complications during the hospitalization of the two groups of patients, and no new symptomatic pulmonary embolism occurred in the two groups. The most common complication was bleeding; six patients in the LCS group developed subcutaneous ecchymosis, puncture site oozing or hematuria, and the bleeding improved after symptomatic treatment; three patient in the ART group developed hemoglobinuria on the day after surgery, which disappeared after treatment such as alkalization of urine. The incidence of complications between the two groups was not statistically significant (*P* > 0.05). The comparison of the complication rate of the two treatment methods is shown in Table 4.

**Table 4:** Comparison of the incidence of complications in the two treatment methods.

**Group**	**New symptomatic PE**	**Bleeding (*n*/%)**	**Hb decrease rate (g/L)**
LCS	0	6 (18.75%)	6.84 ± 14.49
ART	0	3 (9.09%)	5.27 ± 18.02
*t* or χ^2^	-	0.59	0.387
*P*-value	-	0.442	0.700

### Follow-Up

62 of the 65 patients were completely followed up, with 31 patients (91.67%) were followed up in the LCS group, and 31 patients (90.90%) were followed up in the ART group. The average follow-up time was (14.69 ± 7.72) months. During the follow-up period, the patients mainly showed heaviness of the affected limb and anterior tibial edema. One patient in the ART group developed recurrent swelling of the affected limb in 4 months after the operation. The ultrasound indicated a recurrence of thrombosis. After CDT treatment, the patient's symptoms improved and the swelling of lower limbs disappeared. There was no thrombosis on ultrasound. One patient in the LCS group had skin ulceration in the calf, and the ulcer healed after re-intervention.

## Discussion

The inferior vena cava filter can reduce the incidence and mortality of lower extremity DVT-related PE (2), but a study published by the US Food and Drug Administration (FDA) in 2011 showed that the long-term use of the inferior vena cava filter may increase the risk of thrombosis in the filter (7). Joshua D. Kuban conducted a multi-center study on 1,78,327 patients and showed that the placement of the inferior vena cava filter during hospitalization resulted in acute DVT (Inferior Vena Cava Filter Thrombosis, IVCFT) and the proportion of secondary PE (17.6 vs. 11.8%, *P* < 0.001) increased (8). According to the conclusions of a retrospective study of 1,718 cases conducted by Iftikhar Ahmad, the vast majority of patients showed asymptomatic thrombosis after filter implantation. Such thrombosis is often found during the follow-up process by lower limb venous CTV examination, but severe IVCFT may become obstruction of the inferior vena cava. Severe cases can lead to the occurrence of PTS, and even risk of gangrene and amputation (9). Therefore, early and effective removal of filter related inferior vena cava-iliac vein thrombosis plays an important role in shortening the length of stay and reducing complications caused by the filter.

The American College of Chest Physicians (ACCP) guidelines point out that deep vein thrombosis of the lower extremities can be treated with conservative anticoagulation (10). About half of the patients undergo anticoagulation therapy and the thrombus will be reduced or disappear. For patients with symptomatic IVCFT, due to the large secondary thrombotic load of the filter, the effect of simple anticoagulation therapy is not so well. At present, CDT has been widely used in the treatment of DVT. As early as 1994, an RCT conducted by Semba CP showed that the success rate of CDT in the treatment of inferior vena cava-iliac vein thrombosis can reach to 85% (11). However, the high benefit of CDT will increase the risk of local and systemic bleeding. In addition, due to multi-factorial effects, there are still some patients with CDT that cannot effectively eliminate IVCFT. The study by Vedantham showed that for IVCFT patients who failed CDT treatment, the use of PMT can effectively reduce the volume of thrombus, and the immediate thrombus clearance rate can reach to 62% (12). Our study analyzed the therapeutic effects of ART and large lumen catheter suction in filter-related inferior vena cava-iliac vein thrombosis. After the two treatments, 91.3% of the patients achieved thrombus clearance grade II/III, and the clinical symptoms were effectively alleviated; the thrombus clearance rate of the ART group and the LCS group was 67.4 and 86.7%. Both of them are better than Vedantham's use of the Amplatz thrombectomy device for LCS. This may be related to our center's continuous adjustment of the catheter tip direction during the PMT process to improve the efficiency of thrombus removal.

The mainstream percutaneous mechanic thrombectomy procedures include LCS and ART device to remove thrombosis. LCS mainly uses manual suction to form negative pressure, which can physically suck the thrombus. ART device takes both thrombus suction and drug perfusion into account, and uses fluid mechanics to suck the thrombus while spraying the thrombolytic drug to break the thrombus. For patients with inferior vena cava-iliac vein thrombosis who have failed CDT treatment and have contraindications to thrombolysis, PMT is an effective method for removing thrombosis.

During the treatment, no serious complications occurred in the two groups of patients, and the main bleeding areas were subcutaneous and puncture points. Transient hemoglobinuria occurred in the ART group, which may be caused by physical suction that destroys the red blood cell membrane, and the broken red blood cells are excreted by the kidneys. The symptoms disappeared after treatment such as alkalization of urine, and no serious renal damage occurred. Vedantham reported two rare non-hemorrhagic complications. A patient developed partial peroneal nerve palsy during treatment, which is believed to be related to compartment syndrome; after conservative treatment, the patient's symptoms relieved within a few weeks. Another patient developed contralateral iliac vein thrombosis after LCS, which was relieved by thrombolytic therapy (12). There is no report of PMT causing intracranial or gastrointestinal hemorrhage, symptomatic pulmonary embolism or renal failure.

The mechanism of post-thrombotic syndrome caused by deep vein thrombosis of lower extremities has not been fully elucidated. Studies have reported that thrombosis causes chronic venous valve insufficiency, which affects hemodynamics. 33–59% of DVT patients have venous valve dysfunction, and the time of venous recanalization in the segment of valve dysfunction is 2.3 to 7.3 times than that of preserved valve function (13). The follow-up results of our center showed that patients with ART and LCS had only 1 case of post-thrombotic syndrome-related symptoms during the follow-up process, which was lower than the proportion of PTS after the use of anticoagulation therapy alone. It may be related to the effective removal of thrombus in the early stage and maximum protection of valve function. However, there are still some limitations in the evaluation of the impact of valve function during surgery, and a unified evaluation norm and standard has not yet been formed. According to the 2016 epidemiological study of the Jewish General Hospital in Montreal, Canada, the use of CDT+PMT therapy can reduce the risk of PTS by 26% compared with anticoagulation therapy alone, while the risk of bleeding increases by 3% (14). In the long term, IVCFT patients can benefit from the treatment of PMT and reduce the occurrence of PTS. Compared with the traditional catheter contact thrombolysis method, LCS has the advantage of higher treatment success rate and can significantly reduce the risk of PTS (4).

In summary, the technologies of ART and LCS are safe and effective in the treatment of inferior vena cava filter-related thrombosis. The equipment required for LCS is relatively simple, which can be more widely used and promoted in primary hospitals and clinics. ART can reduce the use of thrombolytic drugs and has the advantage of high thrombus clearance, but the cost of equipment hinders its development to a certain extent. This study is a single-center retrospective study, with limited sample size, short follow-up period, and artificial judgment bias for thrombus clearance. We will improve it in future research and work.

## Data Availability Statement

The original contributions presented in the study are included in the article/supplementary material, further inquiries can be directed to the corresponding author/s.

## Ethics Statement

The study was approved by the Ethics Committee of our hospital (KY20210609-15) and by the written consent of patients.

## Author Contributions

XH and JK designed, guided, and funded the study. ZL and GF collected most of the cases and images and drafting of the manuscript. BZ and JG provided a lot of constructive advice in the study. TW and YZ helped with the software calculation. MG assisted with revising pictures. Critical revision of the manuscript and final approval for publication were done by XH, JK, and MG. All authors contributed to the article and approved the submitted version.

## Funding

This work was supported by the National Natural Science Foundation of China (81871463) and the Science and Technology Development Foundation Project of Nanjing Medical University (No. NMUB2019158).

## Conflict of Interest

The authors declare that the research was conducted in the absence of any commercial or financial relationships that could be construed as a potential conflict of interest.

## Publisher's Note

All claims expressed in this article are solely those of the authors and do not necessarily represent those of their affiliated organizations, or those of the publisher, the editors and the reviewers. Any product that may be evaluated in this article, or claim that may be made by its manufacturer, is not guaranteed or endorsed by the publisher.
